# Older People Going Online: Its Value and Before-After Evaluation of Volunteer Support

**DOI:** 10.2196/jmir.3943

**Published:** 2015-05-18

**Authors:** Ray B Jones, Emily J Ashurst, Jo Atkey, Barbara Duffy

**Affiliations:** ^1^Plymouth UniversitySchool of Nursing and MidwiferyPlymouthUnited Kingdom; ^2^RoutewaysPlymouthUnited Kingdom; ^3^Age UK PlymouthPlymouthUnited Kingdom

**Keywords:** digital inclusion, health informatics, social return on investment, older people

## Abstract

**Background:**

Although Internet usage can benefit older people by reducing social isolation, increasing access to services, and improving health and well-being, only a minority are online. Barriers to Internet uptake include attitude and a lack of knowledge and help. We have evaluated volunteer support in helping older people go online. Knowing what value the Internet has been to older people who have just gone online should guide how it is “sold” to those remaining offline.

**Objective:**

Objectives of this study are (1) to assess the feasibility of recruiting volunteers aged 50 years and older and supporting them in helping people (ie, beneficiaries) aged 65 years and older go online, (2) to assess the impact of beneficiaries using the Internet on contacts with others, loneliness, and mental health, and (3) to assess the perceived value to beneficiaries of going online.

**Methods:**

Beneficiaries received help in using the Internet from 32 volunteers in one of two ways: (1) one-on-one in their own homes, receiving an average of 12 hours of help over eight visits, or (2) in small group sessions, receiving 12 hours of help over six visits. We assessed, at registration and follow-up, the number of contacts with others, using Lubben’s 6-item Lubben Social Network Scale (LBNS-6), loneliness, using De Jong Gierveld’s 6-item De Jong Gierveld loneliness scale (DJG-6), and mental well-being, using Tennant’s Short Warwick-Edinburgh Mental Well-Being Scale (SWEMWBS). We also assessed how beneficiaries valued going online using a Social Return on Investment (SROI) approach by postal survey.

**Results:**

A total of 144 beneficiaries were recruited with the aim of helping them go online via one-on-one (n=58) or small group (n=86) sessions. Data through to follow-up were available on 76.4% (110/144) of participants. From baseline to follow-up, the number of contacts with others was significantly increased—LBNS-6, mean 13.7 to mean 17.6—loneliness scores were reduced—DJG-6, mean 2.38 to mean 1.80—and mental well-being improved—SWEMWBS, mean 24.06 to mean 24.96. Out of six options, beneficiaries valued better communication with family and friends most and better health care least as a benefit of using the Internet. Out of nine options, having the Internet was valued less than having TV, but more than, for example, having a weekly visit from a cleaner. There were no associations between values placed on Internet use or volunteer help and psychological improvements.

**Conclusions:**

Volunteer help to go online seemed to result in increased social contacts, reduced loneliness, and improved mental well-being and was valued quite highly by beneficiaries. Although the use of the Internet for health care was the least valued, improved social contact can improve health. Contacting family is likely to be the best “selling point” of the Internet for older people.

## Introduction

### Background

Governments and others are concerned about the digital divide and are funding initiatives to try to close the gap [1]. Age is the biggest predictor of not using the Internet in the United Kingdom (UK). In 2011, only 33% of those aged 65 years and older used the Internet compared to 99% of those aged 14 to 17 [2]. The barriers to Internet uptake include attitudes towards technology, limited experience, lack of physical access, confidence or self-efficacy, knowledge, and social help [3-6].

Studies have shown that older people can benefit from using the Internet. In particular, using the Internet for communication (eg, email) and facilitating contact with friends and family is associated with reduced feelings of social isolation [7-10]. Using the Internet for communication and information is associated with improved general mental well-being and self-efficacy [11,12]. Online access to an increasing range of goods and services (eg, online banking, home delivery, and information) can provide more opportunity to live independently [8,13]. The Internet is, of course, also used for health purposes, including providing online information, peer support, communication with health care professionals, access to medical records, and for repeat prescriptions, as this journal regularly demonstrates.

On the other hand, for some people, emphasis on technology may be the “final straw” in their alienation from modern culture [14], so we need to carefully consider whether, and how, to engage older people in using the Internet. Furthermore, there are many ways to tackle isolation and loneliness [15], and promoting Internet access may not be an obvious choice.

Assuming there are still many older people who are open to the possibility of using the Internet, what would persuade them to go online? Helsper and Reisdorf [4] found that the oldest age group was more likely to indicate a lack of interest and skills as the reason for nonuse. Lack of interest in, or having no need for the Internet was also the main reason cited in 2013 by UK households [16] and in a Scottish study [17].

Although factors such as help-seeking behaviors [18] and demographics [19] may influence the uptake of Internet use, Rogers’ theory suggests that knowing what value an innovation—such as the Internet—has been to other older people who are now online, is a necessary component of discussion with those remaining offline [20]. Older people who have recently gone online are more likely than longer-term users to be like those who continue to be nonusers.

Various projects have sought to address the digital divide through social marketing, training at local centers [21], providing Internet access points at local libraries [22,23], and various community projects, for example, the health communication project by Chu et al [24]. Conducting training and support sessions with older people may improve self-reported measures of Internet confidence, knowledge, and self-efficacy, which can ultimately lead to increased Internet use [23]. Such tuition may be more effective if received from peers of similar age [25].

We have assessed the impact on loneliness, well-being, and perceived value of using the Internet in a project in which peer volunteers helped older people go online.

### Aims

This study had the following three aims:

1. To assess the feasibility and workloads of recruiting volunteers aged 50 years and older, and to support them to help people (ie, beneficiaries) aged 65 and older go online.

2. To assess the impact of using the Internet on contact with others, loneliness, mental health, life satisfaction, and independence using standardized measures.

3. To assess how beneficiaries perceived the value of going online.

## Methods

### Context

Plymouth SeniorNet (PSN) was set up in 2012, funded by the UK’s Big Lottery as part of the Silver Dreams program of interventions to help older people [26]. The estimated population of Plymouth residents aged 65 and older who were non-Internet users was 28,000.

### Ethics

The evaluation project was approved by Plymouth University Faculty of Health, Education, and Society Ethics Committee (28/1/13).

### Interventions

There were two main interventions—volunteers supported beneficiaries (1) one-on-one in their own homes, spending an average of 12 hours together over eight visits, and (2) in 90 small groups spending 12 hours with participants per group. Participants thought to be more physically isolated as determined by PSN were allocated to one-on-one support. Sessions in both settings covered basic computer use, how to get online and search the Internet, online shopping, email, Skype or FaceTime, and online news and entertainment. Volunteers supported some participants in choosing and setting up equipment and broadband. PSN supplied 9 participants with computers on long-term loan agreements and 3 participants received support with broadband costs. The elapsed time for the intervention depended on an agreement between volunteers and beneficiaries about whether they had had sufficient support.

### Recruitment of Volunteers

A total of 36 people responded to local advertisements expressing an interest to become PSN volunteers. Their mean age was 63 years (SD 8.13, range 49 to 84), and most were male (26/36, 72%) and white British (35/36, 97%). Two-thirds (21/33, 64%) had prior volunteering experience and two-thirds (17/27, 63%) used the Internet many times a day. Out of 36 respondents, 4 of them (11%) dropped out during the recruitment process, 31 (86%) proceeded to provide computer support to beneficiaries, and 1 (3%) to manage a PSN online forum. Volunteers attended an introductory training session, which included project overview, an explanation of available roles, safeguarding, and lone working. They were also invited to four quarterly networking events and three technical training sessions.

### Recruitment of Beneficiaries

Awareness of the project was raised through existing contacts with older people through Age UK—UK's largest charity for older people—tenants of Plymouth Community Homes, advertisements in community newspapers and on bus shelters, attendance at other local events, and other personal contacts. Beneficiaries were recruited by referral from Age UK Plymouth to PSN.

### Data Collection

The PSN team documented recruitment, the intervention process, support, and training. Volunteers recorded the number, duration, and content of sessions with beneficiaries. Data from beneficiaries were collected by self-completed questionnaires with assistance where needed. Costs related to PSN staff activities were estimated over the duration of the PSN project and used to model an extension of the project to help 3000 older people get online in Devon, Cornwall, and Somerset, UK.

Baseline questionnaires included demographic data, use of health and social care services, the 6-item Lubben Social Network Scale (LBNS-6) [27]—range 0 to 30, where a high score indicates more connection—the 6-item De Jong Gierveld loneliness scale (DJG-6)—range 0 to 6, where a high score indicates more loneliness [28]—the Short Warwick-Edinburgh Mental Well-Being Scale (SWEMWBS)—range 7 to 35, presenting both raw scores and metric (converted) scores, where a high score indicates better well-being [29-31]—life satisfaction [32], independence using a question from the Investigating Choice Experiments for the Preferences of Older People CAPability measure (ICECAP) index—range 1 to 4, where a high score indicates more independence [33]—and questions from the Personal eHealth Readiness Questionnaire (PERQ) [3]. The first follow-up questionnaire was administered to beneficiaries within a few weeks of their volunteer help coming to an end, which varied between 5 and 42 weeks after completing the baseline questionnaire due to varying periods of volunteer support. It repeated questions from the baseline questionnaire with additional self-reported effects of the intervention on contacts with family and friends.

A second follow-up questionnaire (see Multimedia Appendix 1), a Value of Participation postal survey, which followed the principles of the Social Return on Investment (SROI) [34,35] approach and, in particular, Scholten’s Value Game [36], collected data to evaluate the “worth” to beneficiaries of having computer and Internet tuition. The questionnaire was developed with a small group of older people and volunteers. For each of the four questions in the survey, beneficiaries were asked to distribute 100 tokens based on personal value between a range of activities, at least some of which would be relevant to each participant. We wanted to know about (1) being on the Internet, and (2) getting help to be on the Internet so that we could, to some degree, differentiate between the perceived value of contact with the volunteer and the perceived value of being on the Internet. Question 4 addressed being on the Internet and question 3 addressed receiving help to start using the Internet. The survey was posted simultaneously to all beneficiaries in January 2014. This varied from 1 to 44 weeks—mean of 11 weeks—after completing the follow-up questionnaires.

### Analysis

We explored changes in five main self-reported outcomes from baseline to follow-up by intervention group, gender, age, length of follow-up, and prior Internet connection by paired *t* tests and general linear models. In the general linear models, differences in scores from baseline to follow-up were modelled on intervention, gender, and previous Internet connection (ie, fixed factors), as well as on age and length of follow-up (ie, covariates). This was undertaken with and without the baseline value of the outcome as covariate. We examined both the values placed on Internet use and PSN volunteer help as the raw score and grouped raw scores as more or less than the median compared with improvements in the five main outcomes, and grouped them by intervention group using *t* tests and logistic regression.

## Results

### Beneficiaries Recruited

**Figure 1:**
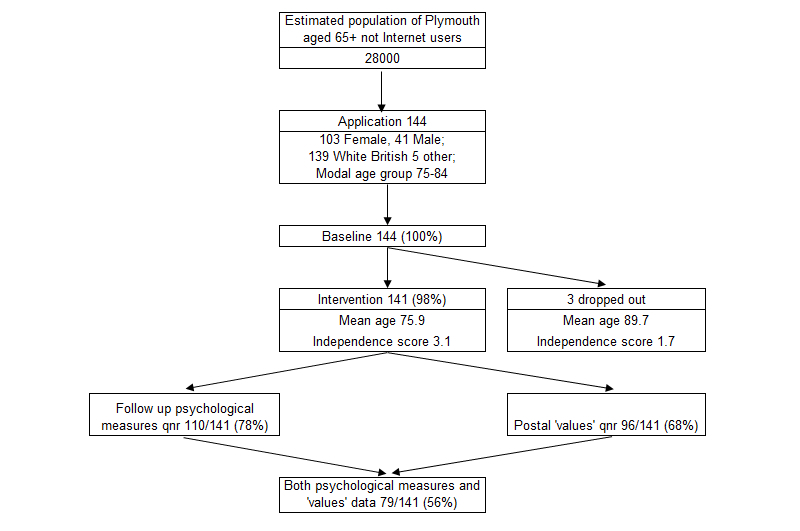
Participant recruitment and dropout.

In total, 144 older people living in the Plymouth area with an interest in learning how to use the Internet were recruited to be supported by home (58/144, 40.3%) or small group (86/144, 59.7%) sessions. Although the target age group was 65 years and older, 14 (9.7%) younger beneficiaries aged 57 to 64 registered and, rather than be turned away, were included. Typically, beneficiaries were aged 75 to 84, were female (103/144, 71.5%), and white British (139/144, 96.5%), which reflected the ethnicity of the older population of Plymouth (see Figure 1). Of the 144 participants, 3 (2.1%) did not receive tuition due to health reasons, leaving 141 (97.9%) who received Internet help. Of the 141 remaining participants, 11 (7.8%) did not take part in the evaluation after the intervention due to health or personal reasons, and 20 (14.2%) did not respond to the follow-up questionnaire requests. Psychological data through to follow-up were, therefore, available on 78.0% (110/141) of beneficiaries, with missing values on some variables. The 3 participants who dropped out were older than the 141 who participated in the intervention (*t*_142_=2.9, *P*=.004) and less independent (*t*_135_=3.5, *P*=.001). The 96 out of the 141 participants (68.1%) who returned the Value of Participation postal survey were more likely to have had the one-on-one home intervention—81.0% home versus 59.0% group, χ^2^_1_=7.6, *P*=.006—and were older—77.5 (SD 8.2) home versus 72.6 (SD 6.7) group, *t*_139_=3.5, *P*=.001—than the 45 (31.9%) who did not complete that questionnaire. There were no other differences by psychological or demographic characteristics at baseline for the groups shown in Figure 1.

The 58 beneficiaries due to be helped at home had been classified as isolated by PSN, based on who they lived with, how easily they could get out and about, and their contact with others. These were older participants and were more likely to have a disability and home help compared to the 86 not considered to be isolated, and who were due to be helped in group settings (see Table 1). All but one of those seen in group settings said they could leave the house unsupported, whereas nearly half of the isolated participants needed support. However, from the standardized questionnaires at baseline, the home-based participants had similar strengths of social networks as the group participants, were not lonelier, had similar mental well-being, and similar satisfaction with life, but were slightly less independent. Those classified as isolated were less likely to see economic barriers to using the Internet for health.

**Table 1:** Comparison of beneficiaries receiving one-on-one help at home with those due to receive group help.

Characteristics	One-on-one help (n=58), mean (SD) or n (%)	Group help (n=86), mean (SD) or n (%)	Test of difference	*P*
Age in years, mean (SD)	79.0 (7.5)	74.3 (8.2)	*t*_142_=3.4	.001
Disability, n (%)	43 (74)	45 (52)	χ^2^_1_=6.9	.008
Home help, n (%)	43 (74)	33 (40) (3 MV^a^)	χ^2^_1_=16.2	<.001
Can leave the house unsupported, n (%)	36 (62)	85 (99)	χ^2^_1_=34.9	<.001
Social network [28] LBNS-6^b^, mean score (SD)	12.3 (6.3)	14.0 (6.8)	No difference	N/A^c^
Loneliness scale DJG-6^b^[29], mean score (SD)	2.4 (1.6) (2 MV)	2.4 (1.7) (18 MV)	No difference	N/A
Mental well-being SWEMWBS^b^, mean score (SD)	24.0 (3.6) (2 MV)	24.0 (5.3) (13 MV)	No difference	N/A
Satisfaction with life [32], mean score (SD)	7.1 (2.0)	7.6 (2.4) (2 MV)	No difference	N/A
Independence [33], mean score (SD)	3.0 (0.7)	3.2 (0.8) (7 MV)	*t*_132_=1.98	.05
Perceived economic barriers to using the Internet for health [3], mean score (SD)	3.55 (1.7)	2.44 (1.6)	*t*_142_=3.9	<.001

^a^Missing values (MV).

^b^6-item Lubben Social Network Scale (LBNS-6), 6-item De Jong Gierveld loneliness scale (DJG-6), Short Warwick-Edinburgh Mental Well-Being Scale (SWEMWBS).

^c^Not applicable (N/A).

Just under half (61/138, 44.2%) of the beneficiaries had never used the Internet, a third (45/138, 32.6%) had used it a few times, and 23.2% (32/138) claimed to have used it fairly often, although not in the last 3 months, prior to the project. More than a third (53/141, 37.6%) had previously attended a computer class and two-thirds (93/138, 67.4%) had asked someone else to use the Internet on their behalf. As all were responding to an offer of help with skills, we might assume (1) that they were interested in trying the Internet, perhaps again, and (2) that skills rather than lack of access or cost may be the main reason for current Internet nonuse [4].

Two-thirds of beneficiaries (95/138, 68.8%) had home Internet connections and three-quarters (105/143, 73.4%) had a home computer. Laptops were the most common device (62/104, 59.6%), followed by desktop computers (19/104, 18.3%), tablets (16/104, 15.4%), and multiple devices, such as a laptop and a tablet (7/104, 6.7%). Most beneficiaries were interested to learn about using email (127/140, 90.7%), followed by Skype (100/140, 71.4%), online shopping (100/144, 69.4%), then games (67/140, 47.9%), and social networking (55/140, 39.3%).

### Intervention Resources and Feedback

In total, volunteers had 974 contacts with 141 beneficiaries—498 contacts in group sessions, 476 contacts in one-on-one sessions—and an average of 6.9 contacts per beneficiary. In total, there were 996 hours of contact with groups and 702 hours of one-on-one contact. The total period (ie, elapsed time) over which volunteers were in contact with beneficiaries, and hence the gap between completing the baseline and follow-up questionnaires, varied considerably from 5 to 42 weeks. Beneficiaries who received one-on-one contact received this over a longer period—mean 18.5 (SD 7.4) weeks—than those attending group sessions—mean 10.7 (SD 4.2) weeks—(*t*_108_=7.0, *P*<.001). Several beneficiaries remarked that they felt less intimidated by the prospect of working with older volunteers.

### Internet Use at Follow-Up

At follow-up, one-fifth (22/107, 20.6%) of beneficiaries reported using the Internet less than once a week, while four-fifths used it quite frequently—14.0% (15/107) used it many times a day, 34.6% (37/107) used it at least once a day, and 30.8% (33/107) used it at least once a week. Of those who had never used the Internet prior to Internet tuition, 68% (32/47) now used it at least once a week, including 28% (13/47) who used it at least once a day and 9% (4/47) who used it many times a day.

Most (100/109, 91.7%) beneficiaries now had a home Internet connection. Of those who did not have an Internet connection at the start of the project, 83% (29/35) did at follow-up, accounting for 30% (29/96) of those who reported having a home connection following tuition. However, 4% (3/70) of those with an Internet connection, initially, terminated their connection after the project.

Most beneficiaries had used the Internet for information (90/108, 83.3%) and email (82/108, 75.9%). The next most frequent use was Skype (42/108, 38.9%). Most beneficiaries accessed the Internet using a home desktop computer or laptop (66/108, 61.1%), followed by a mobile device, such as a tablet (23/108, 21.3%). Only a few reported not having accessed the Internet since the project (6/108, 5.6%).

### Impact of Internet Use

At baseline, more than two-thirds (86/124, 69.4%) of beneficiaries were lonely—DJG-6 score of 0 to 1 [26]—compared to 52% of older people in Spain [37] and New Zealand [38]. However, the baseline mental well-being scores of the Plymouth SeniorNet population were better than the overall scores for all Silver Dreams projects—24.3 versus 21.78 [26].

Simple before-after analysis (see Table 2) suggested that participants had increased social networks, reduced loneliness, and improved mental well-being after the intervention. There was no measurable impact on satisfaction with life or independence scores, even though 46.8% (51/109) reported that involvement in the project had made a difference to their satisfaction with life, and most of those with limited independence at baseline reported an improvement (13/17, 76%) at follow-up.

**Table 2:** Mean scores and significance of score changes from baseline to follow-up for up to 110 participants for various measurements by intervention and for all participants.

Scale			Scores, mean (SD)		Mean change	n	*t* ^a^	*df*	*P*	Impact of intervention
			Baseline	Follow-up						
**One-on-one sessions** **(n=45)**										
	Contacts^b^		12.25 (6.18)	13.82 (6.17)	1.57	44	2.05	43	.05	Increased social networks
	Loneliness^c^		2.42 (1.66)	2.34 (1.64)	-0.08	41	0.30	40	.76	No difference
	**Mental well-being** ^d^									
		Raw score	26.90 (3.49)	27.64 (3.28)	0.74	39	1.32	38	.19	No difference
		Metric score	24.30 (3.35)	24.95 (3.16)	0.65	39	1.15	38	.26	No difference
	Satisfaction^e^		7.09 (2.11)	7.16 (2.35)	0.07	45	0.17	44	.86	No difference
	Independence^f^		3.00 (0.62)	2.95 (0.65)	-0.05	43	0.50	42	.62	No difference
**Group sessions** **(n=65)**										
	Contacts		15.03 (6.42)	16.69 (5.57)	1.66	65	3.50	64	.04	Increased social networks
	Loneliness		2.35 (1.65)	1.37 (1.43)	-0.98	51	3.39	50	.001	Reduced loneliness
	**Mental well-being**									
		Raw score	25.96 (5.73)	27.63 (2.97)	1.67	54	2.19	53	.03	Improved mental well-being
		Metric score	23.89 (4.91)	24.97 (3.18)	1.08	54	1.68	53	.10	No difference
	Satisfaction		7.60 (2.33)	8.14 (1.22)	0.54	63	2.00	62	.05	Improved satisfaction
	Independence		3.27 (0.76)	3.29 (0.59)	0.02	59	0.16	58	.87	No difference
**All participants** **(n=110)**										
	Contacts		13.91 (6.44)	15.53 (5.56)	1.62	109	2.87	108	.005	Increased social networks
	Loneliness		2.38 (1.64)	1.80 (1.59)	-0.58	92	2.92	91	.004	Reduced loneliness
	**Mental well-being**									
		Raw score	26.36 (4.92)	27.63 (3.09)	1.27	93	2.56	92	.01	Improved mental well-being
		Metric score	24.06 (4.31)	24.96 (3.16)	0.90	93	2.04	92	.04	Improved mental well-being
	Satisfaction		7.39 (2.24)	7.73 (1.83)	0.34	108	1.53	107	.13	No difference
	Independence		3.16 (0.71)	3.15 (0.64)	-0.01	102	0.13	101	.89	No difference

^a^Paired *t* tests.

^b^Number of contacts measured with the 6-item Lubben Social Network Scale (LBNS-6) [27].

^c^Loneliness measured with the 6-item De Jong Gierveld loneliness scale (DJG-6) [28].

^d^Mental well-being measured with the Short Warwick-Edinburgh Mental Well-Being Scale (SWEMWBS) [29-31].

^e^Satisfaction with life [32].

^f^Independence as in Coast et al [33].

When viewed by intervention type, the pattern of change was consistent with the overall change in these five variables (see Table 2). In paired *t* tests, those in the group intervention showed a greater reduction in loneliness—0.98 group versus 0.07 home, *t*_90_=2.41, *P*=.018—but not in the other four main outcomes, compared to those in the one-on-one intervention.

Modelling changes in the five outcomes by demographics, length of follow-up, prior Internet connection, and intervention group, did not identify any major predictors (see Table 3). However, in all cases the baseline value of the outcome was a significant predictor at *P*<.01, suggesting that all improvements seen may just be “regression to the mean.” Length of follow-up was a significant predictor of increased connections (*F*_*109*_=5.21, *P*=.025) and intervention type was a predictor of increased independence (*F*_*140*_=4.75, *P*=.03).

**Table 3:** Significant predictor variables in general linear models predicting improvement in five outcome variables.

Improved outcome	Predictors	
	Not including baseline as covariate	Including baseline as covariate
Contacts	No predictors	Baseline value (*P*<.001) Length of follow-up (*P*=.03)
Loneliness	Intervention*previous connection (*P*=.04)	Baseline value (*P*<.001)
Mental well-being (metric)	No predictors	Baseline value (*P*<.001)
Satisfaction	No predictors	Baseline value (*P*<.001) Intervention*gender (*P*=.05)
Independence	No predictors	Baseline value (*P*<.001) Intervention (*P*=.03)

### Value of Participation

Of the 141 beneficiaries, 101 (71.6%) responded to the Value of Participation postal survey but 5 (3.5%) returned incomplete questionnaires—1 participant was ill, 1 had died, 1 was no longer using the Internet, 1 was dissatisfied with the intervention and did not want to complete the questionnaire, and for 1 the reasons were unknown. Data from this survey were, therefore, available for 68.1% (96/141) of the beneficiaries (Figure 1).

Better communication with family and friends was ranked highest out of six options by beneficiaries as a benefit from using the Internet (mean 35.63, SD 21.60), followed by being entertained or stimulated (mean 21.96, SD 22.80), and feeling more confident because of their newly learned skills (mean 18.18, SD 18.95) (see Table 4). Better health care was seen as the least important. Beneficiaries also thought that the tuition they received had also benefited their family and friends, most notably better communication with them was ranked highest (mean 49.50, SD 28.38) out of four options (see Table 5). Receiving help from a PSN volunteer in using the Internet was ranked highest out of nine options in terms of value to them (mean 21.55, SD 20.93). This was followed by receiving a phone call each week from a family member or friend (mean 20.21, SD 16.96), and being able to get out and about by themselves (mean 14.54, SD 15.65) (see Table 6). Finally, giving up the Internet for 1 week was ranked second highest (mean 16.49, SD 15.89), following giving up their TV for a week (mean 23.63, SD 16.75) out of nine options of things they did not want to give up (see Table 7).

Those who had one-on-one tuition at home were more likely to value the PSN volunteer than those helped in group settings—25.89 (SD 19.98) home versus 17.39 group (21.18), *t*_94_=2.0, *P*=.046—but there was no difference in the value placed on being online between the two interventions. There were no associations between values placed and the five main psychological outcomes from logistic regression analysis.

**Table 4:** Values assigned to personal use of the Internet based on answers to question 1 from the Value of Participation postal survey: Have you benefited from using the Internet in any of these ways?

Benefits from using the Internet (n=90)	Score out of 100, mean (SD)
Better communication	35.63 (21.60)
Being entertained or stimulated	21.96 (22.80)
Feeling more confident	18.18 (18.95)
Being more independent	11.18 (14.98)
Saving money or having a better range of goods	7.03 (11.85)
Better health care	6.02 (9.77)

**Table 5:** Perceived value for family members of older person’s Internet use based on answers to question 2 from the Value of Participation postal survey: Have your family or friends benefited from you using the Internet?

Benefits by family or friends from you using the Internet (n=83)	Score out of 100, mean (SD)
Better communication	49.50 (28.38)
Saving money or having a better range of goods	17.69 (19.84)
Being entertained or stimulated	17.40 (21.54)
Not having to do things for me	15.41 (20.79)

**Table 6:** Help using the Internet compared to other activities based on answers to question 3 from the Value of Participation postal survey: How much are the following activities worth to you?

Internet help and other activities (n=96)	Score out of 100, mean (SD)
Receiving help from a Plymouth SeniorNet volunteer	21.55 (20.93)
Having a phone call from my family/friend each week	20.21 (16.96)
Being able to get out and about on my own	14.54 (15.65)
Having someone clean my house/flat	13.82 (18.36)
Being taken out to a nice pub in the country for lunch	7.83 (10.20)
Getting a letter or postcard from my family/friend	7.53 (10.77)
Spending an afternoon enjoying the garden	6.92 (10.17)
Having someone help sort out bills, investments, or finance	4.38 (8.62)
Having someone cook me lunch at home	3.24 (7.75)

**Table 7:** Value of keeping Internet access compared to other activities based on answers to question 4 from the Value of Participation postal survey: Which of these do you really not want to give up?

Continued Internet access and other activities (n=95)	Score out of 100, mean (SD)
TV for a week	23.63 (16.75)
Internet for a week	16.49 (15.89)
Bus pass for a week	15.70 (18.65)
Reading the newspaper for a week	9.78 (10.97)
One weekly visit from my cleaner	9.53 (13.90)
One weekly tea and biscuits with a friend	7.61 (10.76)
A social event for a week (eg, bowling, bridge, pub)	7.14 (10.39)
One weekly visit from my gardener	5.50 (9.92)
Going to church for a week	4.63 (9.14)

### Costs

PSN received funding of £172,000. Setup and learning costs to get the project started were estimated as £50,000. Costs for taster sessions—105 people not reported in this paper—were estimated as £20,000. Therefore, the cost to help 144 people online was £102,000, or £708 per person. This did not include the costs of volunteers, but if we assume that volunteers benefited themselves from their activity we might exclude those costs. Based on workloads for PSN and how these might have scaled up—with economies of scale—in a 4-year project for the largely rural counties of Devon, Cornwall, and Somerset, UK, we estimated a project cost of £1 million to help 3000 people, or £333 per person.

### Social Return on Investment

Having the Internet is valued by participants more than items such as cleaning and meals out that can be valued at £20 to £25 per week (see Table 6). An annual value for being online might, therefore, be £1000 to £1300. An intervention such as PSN would, therefore, “pay for itself” in under a year.

## Discussion

### Principal Findings

The longer-term aim of PSN was that going online would help older people be better connected with their peers, family, and sources of support, have a greater sense of control in their lives through the use of computers, have a greater sense of belonging to a community, be able to use information technology to improve health and well-being, and build resilience to manage change effectively. The indications from the before-after scores for social network, loneliness, and mental well-being for the beneficiaries, along with the value they placed on using the Internet for communication suggest that helping older people to use the Internet is of considerable value. Social isolation has mortality risks as great as smoking and obesity [39,40]. Reducing social isolation is, therefore, a health issue, but may not be seen as such either by older people themselves or by policy makers.

The PSN intervention of recruiting people aged 50 years and older to help those aged 65 years and older get online seemed to work well. Volunteers were relatively easily recruited and retained, however the project itself terminated with the end of Lottery funding for the supporting PSN team, although some volunteers may have continued informally to help older people go online. Some PSN volunteers transferred to Age UK Plymouth as IT trainers/facilitators after the project ended. PSN funding had been substantial, but a large proportion of these funds were used in setting up the project and learning what worked in establishing the recruitment and training of volunteers and recruitment of beneficiaries. This method of getting people online is still relatively expensive compared to some other methods, such as group drop-in sessions. However, that some people needed support over quite long periods (ie, up to 42 weeks) suggests that assumptions that older people can become Internet users after one or two sessions may be overoptimistic.

We demonstrated two approaches to help older people get online—one-on-one and group sessions—but it is difficult to compare the two interventions, as allocation was based on perceived physical ability to get out of the house and, thereby, to attend group sessions. Those given one-on-one help were older, more likely to be disabled, and needed home help so it may not have been feasible for them to attend group sessions. However, those attending group sessions seemed, on simple paired *t* test analysis, to have a greater reduction in loneliness compared to those in one-on-one sessions.

One strength of the PSN approach may be the support offered by volunteers on practical aspects of equipment and getting online, for example, being there to oversee the installation of broadband and to set up new equipment. On joining PSN, two-thirds of beneficiaries already had Internet connections but were not sure how to use them and had not used the Internet in the previous 3 months. Although this was higher than in a cross-sectional household survey in the same town in which just under half (30/71, 42%) of non-Internet users had an Internet-connected computer at home [3], indicating that participants in that study were more predisposed to try using the Internet, half of non-Internet users may live in Internet-connected households. We did not collect data on bereavement, but it is possible that many non-Internet-using older people had previously relied on a now deceased partner for that “task” within their relationship [41] and now had to take on this new responsibility.

A quarter of the beneficiaries felt their involvement in the project had made a notable difference to the number of contacts with family and friends, in particular the number of relatives they felt close enough to that they could call on for help. This was supported by the Value of Participation survey in which better communication was the biggest benefit to them and their family and friends from learning to use the Internet. Receiving help from a volunteer in using the Internet was highly valued by beneficiaries and rated higher than other activities, such as getting out and about on their own or having a cleaner in to help.

Studies of this sort, including, for example, the overall evaluation of the Silver Dreams project [26], typically use a before-after design and attribute improvement to the intervention. On this same basis, using paired *t* test analysis, scale measures showed a significant decrease in loneliness and increase in mental well-being among beneficiaries—18% indicated that their involvement in the project had made a notable difference to their satisfaction with life. However, a linear model approach that included baseline values of each outcome variable suggested that at least part of this change may be “regression to the mean.”

### Limitations

There was no control group and participants volunteered for help to go online. Therefore, the participants still represented those who were willing to try the Internet. Although our before-after measures may, therefore, be partly explained by a Hawthorne effect, including the social aspects of being involved in the project, there is no reason to believe that their views on the value of being online are invalid.

As participants were chosen for each type of intervention and not randomized, it had not been our a priori intention to compare effectiveness of the two interventions. We assumed before the project that physical independence to get out of the house might be associated with psychological isolation, loneliness, and mental well-being. This proved not to be the case. We, therefore, took the opportunity to examine, post hoc, which approach seemed to work given the baseline psychological status of participants in the two groups. Some will argue that comparisons that were not predefined should not be made.

Fitting a model to the five main psychological outcomes including baseline values suggests that the improvements seen may just be “regression to the mean.” We, therefore, may be overly optimistic in assuming that the PSN intervention had any effect. However, the simple before-after approach has been used for other studies, including from the Silver Dreams program [26], and our method of presentation allows comparison with those studies.

Other studies [4,5] have shown education to be a good predictor of the uptake of use of the Internet. However, we did not collect data on the educational level of beneficiaries and were unable to make such comparisons.

### Recommendations

1. This project has shown that many older people can be helped to get online. They rated the value of being online highly compared to other daily activities, and using the Internet for communication with family was most valued, whereas using the Internet for health was not seen as particularly important. Although there is evidence from elsewhere that many older people are not interested in using the Internet, these findings suggest that campaigns to motivate those older people who are, as yet, not interested in going online should focus on communication with family.

2. Others have tried intergenerational support for older learners [42], but PSN beneficiaries indicated that support by someone closer to them in age was important.

3. Many of the older people we contacted were prepared to buy their own equipment, in particular, tablet computers proved popular. The decreasing cost of tablets suggests that, although cost may be important for some, volunteer support should be the focus.

4. A mix of one-on-one and group sessions is likely to be needed. Those with limited mobility may struggle to attend group sessions out of the home.

## Multimedia Appendix 1

Extension to beneficiary follow-up questionnaire.

## Abbreviations

DJG-6: 6-item De Jong Gierveld loneliness scale
ICECAP: Investigating Choice Experiments for the Preferences of Older People CAPability measure
LBNS-6: 6-item Lubben Social Network Scale
MV: missing values
N/A: not applicable
PERQ: Personal eHealth Readiness Questionnaire
PSN: Plymouth SeniorNet
SROI: Social Return on Investment
SWEMWBS: Short Warwick-Edinburgh Mental Well-Being Scale
UK: United Kingdom
