# Supplementary material for: Older People Going Online: Its Value and Before-After Evaluation of Volunteer Support
Source: J Med Internet Res. 2015 May 18;17(5):e122. doi: 10.2196/jmir.3943 (PMC4468603; doi:10.2196/jmir.3943)
Supplement: Supplementary file 1 [file jmir_v17i5e122_app1.pdf]

RESEARCH WITH PLYMOUTH UNIVERSITY

Plymouth SeniorNet

BENEFICIARY ID NUMBER:

Extension to beneficiary follow-up questionnaire

Today's date……………………………

1. Have you benefited from using the Internet in any of these ways?

(a) ✐ Cross out any of the 6 boxes below that are not relevant to you (i.e. you have not experienced as a result of learning to use the Internet).

(b) ✐ With the <u>remaining boxes only</u>, imagine you have 100 counters. Please share out the 100 counters among the activities below by how much you feel you have benefited from the Internet. More counters means it is worth more to you.

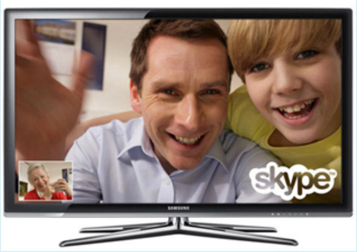

Better communication
with family, friends, or others by email or Skype, or being able to receive photos, or share things with your family or friends

✐ AMOUNT:

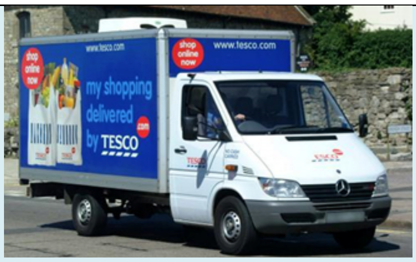

Being more independent
by being able to do something that otherwise someone may have had to help you with, or do for you. I.e. Internet banking, or applying for benefits online, or getting shopping delivered, or finding out about things

✐ AMOUNT:

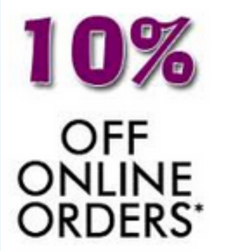

Saving money or having a better range of goods
by shopping online or by finding out about prices and services online

✐ AMOUNT:

BBC iPlayer

Being entertained or stimulated
by finding out facts, or playing games, having access to entertainment online, watching TV on 'catch up', taking part in hobbies, finding out about your family tree

✐ AMOUNT:

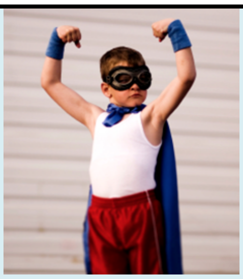

Feeling more confident
because of your new skills and because you have a better idea of what is going on via the Internet and what things are happening

✐ AMOUNT:

Patient access

Better health care
because of things you have learned online, or through having better access to some online health services like online appointment booking, and prescriptions

✐ AMOUNT:

2. Have your family or friends benefited from you using the Internet?

(a) ✎ Cross out any of the 4 boxes below that are not relevant to you (i.e. your family/ friends have not experienced as a result of your learning to use the Internet).

(b) ✎ With the <u>remaining boxes only</u>, imagine you have 100 counters. Please share out the 100 counters among the activities below by how much you feel your family/ friends have benefited from your learning to use the Internet. More counters means it is worth more to your family.

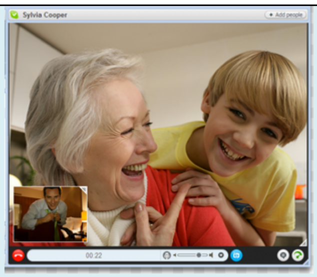

Better communication
with family, friends, means that they can get in touch with you more easily, and maybe have peace of mind.

✎ AMOUNT:

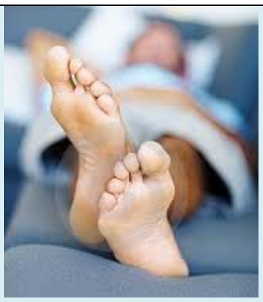

Not having to do things for you
now that you can do these things for yourself on the Internet, e.g. shopping online instead of driving you to the supermarket

✎ AMOUNT:

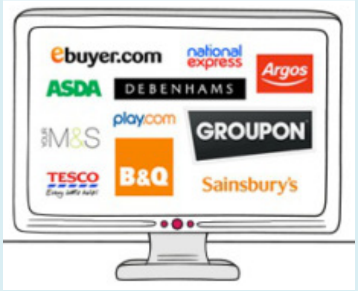

Saving money or having a better range of goods
because you can tell them from information you got from the Internet.

✎ AMOUNT:

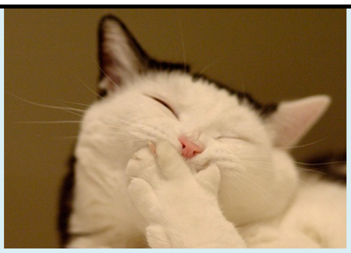

Being entertained or stimulated
because you have found out things, or found entertainment for them, e.g. found information online about a hobby or interest of theirs

✎ AMOUNT:

3. How much are the following activities worth to you?

(a) ✐ Cross out any of the 9 boxes below that are not relevant to you.

(b) ✐ With the remaining boxes only, imagine you have 100 counters. Thinking about what these activities have been worth to you over the last six months, please share out the 100 counters among the activities by how much you would 'spend' to KEEP the activity. More counters means it is worth more to you.

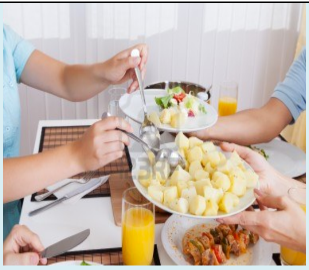

Have someone cook me lunch at home

✐ AMOUNT:

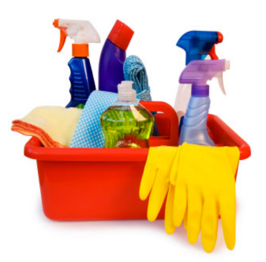

Have someone clean my house/ flat

✐ AMOUNT:

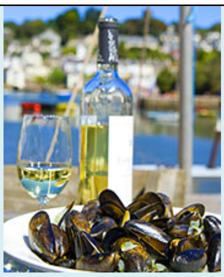

Be taken out to a nice pub in the country for lunch

✐ AMOUNT:

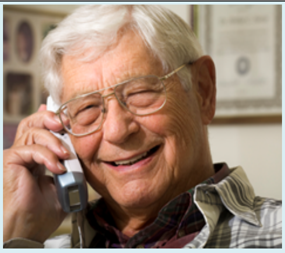

Having a phone call from my family/ friend each week

✐ AMOUNT:

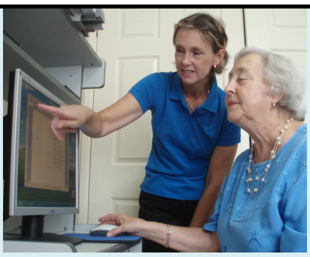

Receive help from a Plymouth SeniorNet volunteer in using the Internet

✐ AMOUNT:

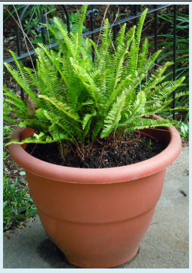

Spend an afternoon pottering in the garden

✐ AMOUNT:

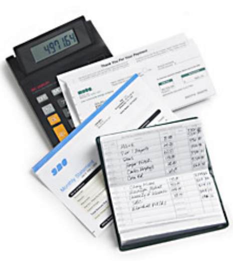

Have someone help sort out bills, investments or finances

✐ AMOUNT:

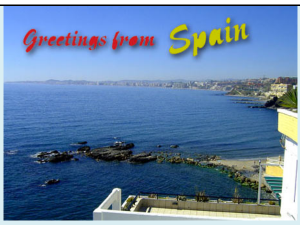

Getting a postcard from my family/ friend

✐ AMOUNT:

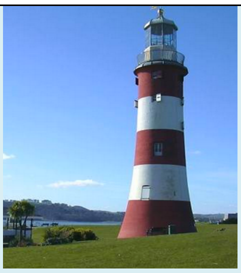

Being able to get out and about (e.g. by the sea, in the country, round the town)

✐ AMOUNT:

4. Which of these do you really not want to give up?

(a) ✐ Cross out any of the 9 boxes below that are not relevant to you.

(b) ✐ With the <u>remaining boxes only</u>, imagine you have 100 counters. Please share out the 100 counters among the activities by how much you value them and want to avoid giving up (i.e. so if you value using your bus pass more than watching TV, you would give more counters to the bus pass). More counters means it is worth more to you.

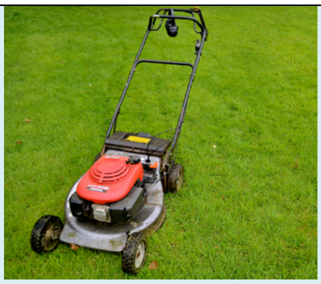

Give up your gardener for a week

✐ AMOUNT:

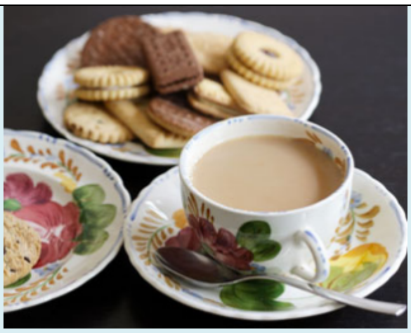

Give up one weekly tea and biscuits with a friend

✐ AMOUNT:

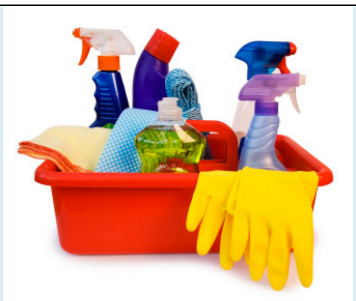

Give up one weekly visit from your cleaner

✐ AMOUNT:

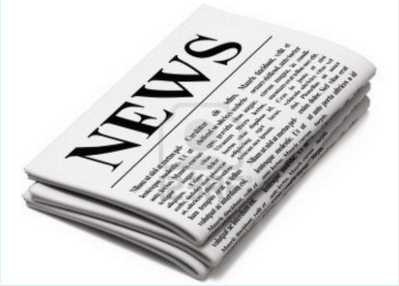

Give up reading the newspaper for a week

✐ AMOUNT:

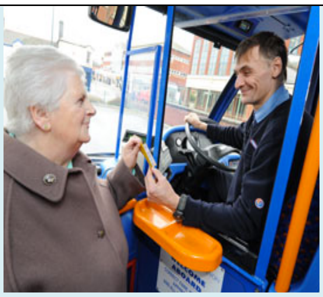

Give up your bus pass for a week

✐ AMOUNT:

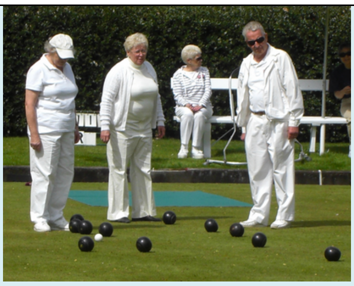

Give up a social event for a week (i.e. bowls/ bridge/ pub)

✐ AMOUNT:

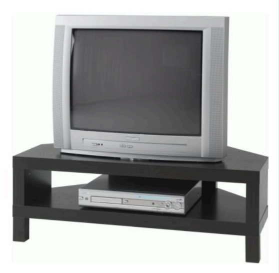

Give up your TV for a week

✐ AMOUNT:

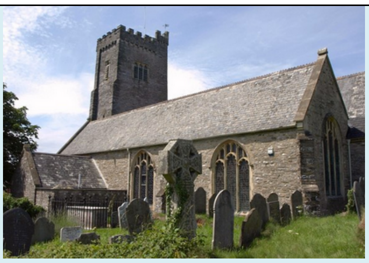

Give up going to church for a week

✐ AMOUNT:

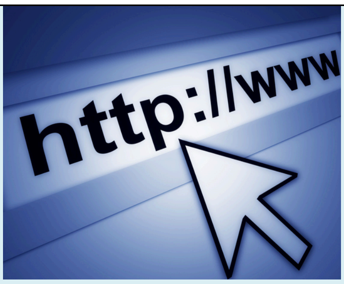

Give up the Internet for a week

✐ AMOUNT:

Thank you for your time completing this questionnaire!
